# Acute Pancreatitis and Diabetic Ketoacidosis following L-Asparaginase/Prednisone Therapy in Acute Lymphoblastic Leukemia

**DOI:** 10.1155/2014/139169

**Published:** 2014-02-10

**Authors:** Dania Lizet Quintanilla-Flores, Miguel Ángel Flores-Caballero, René Rodríguez-Gutiérrez, Héctor Eloy Tamez-Pérez, José Gerardo González-González

**Affiliations:** ^1^Internal Medicine Department, “Dr. José Eleuterio González” University Hospital and School of Medicine, Universidad Autónoma de Nuevo León, Avenida Francisco I. Madero pte. y Avenida Gonzalitos s/n, Colonia Mitras Centro, 64460 Monterrey, NL, Mexico; ^2^Research Division, School of Medicine, Universidad Autónoma de Nuevo León, Avenida Francisco I. Madero pte. y Avenida Gonzalitos s/n, Colonia Mitras Centro, 64460 Monterrey, NL, Mexico

## Abstract

Acute pancreatitis and diabetic ketoacidosis are unusual adverse events following chemotherapy based on L-asparaginase and prednisone as support treatment for acute lymphoblastic leukemia. We present the case of a 16-year-old Hispanic male patient, in remission induction therapy for acute lymphoblastic leukemia on treatment with mitoxantrone, vincristine, prednisone, and L-asparaginase. He was hospitalized complaining of abdominal pain, nausea, and vomiting. Hyperglycemia, acidosis, ketonuria, low bicarbonate levels, hyperamylasemia, and hyperlipasemia were documented, and the diagnosis of diabetic ketoacidosis was made. Because of uncertainty of the additional diagnosis of acute pancreatitis as the cause of abdominal pain, a contrast-enhanced computed tomography was performed resulting in a Balthazar C pancreatitis classification.

## 1. Introduction

The long-term outcome of acute lymphoblastic leukemia (ALL) has improved dramatically during the last few decades because of the development of well-designed and effective treatment protocols. Since 1961, L-asparaginase, combined with danorubicin, vincristine, and prednisone, the cornerstone treatment for ALL. They are used in remission, induction, and intensification phases in all pediatric regimens and in the majority of adult treatment protocols [1, 2]. Long-term, event-free, survival rates in children are currently around 80% and overall survival rates are close to or exceeding 90% of pediatric patients. Although overall survival rates in adults have improved in recent years, only 38% to 50% achieve long-term survival [3].

The most common complications associated with L-asparaginase are abdominal pain and allergic reactions. Other side effects include liver dysfunction, coagulation defects and central nervous system depression. Moreover, acute pancreatitis (AP), hyperglycemia, and diabetic ketoacidosis (DKA) can also be present [4]. Even though these side effects are well known, the combination of both DKA and AP represents unusual conditions generally reported as benign and self-limited [5].

We report a case of a 16-year-old male patient who developed transient diabetes mellitus following L-asparaginase therapy with ketoacidosis and acute pancreatitis as the mode of presentation.

## 2. Case Presentation

A 16-year-old Hispanic male patient was admitted to our hospital. He was diagnosed as having acute lymphoblastic leukemia 5 months earlier. He was during the remission induction phase of therapy with mitoxantrone, vincristine, prednisone, and L-asparaginase, receiving last dose one week prior to his admission. He presented to the emergency department with a history of 3 days of abdominal pain, nausea, vomiting, and anorexia, referring to his last vomiting episode as hematemesis. His blood tests reported venous gasometry resulting with a pH 7.27, sodium bicarbonate 15.7 mmol/L, pCO2 24 mmHg; hemoglobin 12.6 g/dL (12.2–18.1 g/dL), neutrophylia of 9.58 cells/mm^3^ (4.0–11.0 cells/mm^3^); hyperglycemia of 594 meq/L (60–100 meq/L), blood urea nitrogen 25 mg/dL (7–20 mg/dL), creatinine 1.17 mg/dL (0.6–1.4 mg/dL), sodium 122 mmol/L (135–145 mmol/L); urine test with ketones; hyperamylasemia of 476 U/L (30–110 U/L), and a serum osmolarity of 282 mosm/kg. The diagnosis of diabetic ketoacidosis was made.

Despite medical treatment, the patient persisted with abdominal pain and refractory hyperglycemia associated with hyperamylasemia. Because of diagnostic uncertainty a contrast-enhanced computed tomography was performed, resulting in a Balthazar C pancreatitis classification. Furthermore he had a serum lipase test in 825 Units/L, confirming diagnosis of acute pancreatitis additional to the previous diagnosis of diabetic ketoacidosis.

The day after the patient was admitted, he underwent upper endoscopy, resulting in Mallory-Weiss syndrome, explaining his episode of hematemesis. After three days of fluid correction, insulin infusion, fasting, and analgesic therapy, the patient was discharged presenting no further complications.

## 3. Discussion

This case represents a combination of two rare complications related to L-asparaginase treatment during the remission induction phase of treatment in a patient with ALL. Pancreatic toxicity during L-asparaginase treatment has been reported with a prevalence of 16.2%, with an incidence of AP ranging from 2 to 18% [3, 6]. AP results from direct deprivation on the protein metabolism leading to a disturbed synthesis of albumin, fibrinogen, plasma clotting factors IX and X, plasminogen, and antithrombin III, which leads to considerable toxicity particularly to organs associated with high protein production such as liver and pancreas [7]. It can be observed in all phases of ALL, after a median of 5.5 doses of therapy and a median interval from the last dose of L-asparaginase administration of 4–9 days [8]. L-asparaginase AP is generally developed in patients below 10 years of age, and it has been associated with higher doses of L-asparaginase in the chemotherapy regimen [3, 8].

Hyperglycemia occurs in about 10–15% pediatric patients with ALL, associated with the use of L-asparaginase, glucocorticoids, AP, infection, and the leukemic process itself [9]. L-asparaginase induces hyperglycemia via depletion of L-asparagine and as a consequence decrement of insulin synthesis; additionally it decreases insulin secretion from pancreatic *β*-cells, impairs insulin receptor function, and causes hyperglucagonemia [4, 9]. The risk and severity of hyperglycemia increases when L-asparaginase and glucocorticoids are used concomitantly; however it is generally self-limiting resulting in no complications [10]. Additionally, pancreatic toxicity can lead to metabolic alterations such as hyperglycemia and diabetes mellitus in 2.5–23% of patients [11].

Despite the fact that hyperglycemia is commonly associated with L-asparaginase, DKA represents a rare condition with a reported prevalence of 0.8% [9]. Older age, obesity, and Down's syndrome represent the main known risk factors for the development of DKA, with age being the only factor detected in our patient. This increased risk may be due to the association between insulin resistance and increased growth hormone secretion observed during late childhood and adolescence [9, 12]. It is possible that the combination of L-asparaginase, glucocorticoids and AP represented synergistic factors for the development of DKA in our patient.

The clinical course of both conditions can vary from mild to severe disease. A higher mortality rate and a lower median 2-year survival associated with AP during L-asparaginase treatment have been reported [8]. On the other hand, even though overt hyperglycemia can be an independent predictor factor for survival in children with ALL during induction therapy, DKA generally results in short-term morbidity and hospital admission, without the alteration of ALL outcome and modification in the chemotherapeutic regimens [9, 13]. Fortunately, our patient achieved complete resolution of symptoms, normalization of laboratory results, and imaging studies without major adverse events regarding both diseases.

We conclude that the combination of AP and DKA associated with L-asparaginase use are rare complications with significant morbidity and mortality. During therapy with L-asparaginase, we recommend a close monitoring for hyperglycemia as well as acute pancreatitis in order to reduce the adverse events related to both conditions.

## Abbreviations

ALL: Acute lymphoblastic leukemia
AP: Acute pancreatitis
DKA: Diabetic ketoacidosis.
